# Exercise-induced irisin improves follicular dysfunction by inhibiting IRE1α-TXNIP/ROS-NLRP3 pathway in PCOS

**DOI:** 10.1186/s13048-023-01242-x

**Published:** 2023-07-31

**Authors:** Yajing Weng, Yaling Zhang, Daojuan Wang, Rong Wang, Zou Xiang, Shanmei Shen, Hongwei Wang, Xiaoke Wu, Yanting Wen, Yong Wang

**Affiliations:** 1 State Key Laboratory of Analytical Chemistry for Life Science & Jiangsu Key Laboratory of Molecular Medicine, Medical School, Nanjing University, Nanjing, 210093 China; 2 School of Medicine, Jiaxing University, Jiaxing, 314001 China; 3 Department of Pain, Medical School, The Affiliated Drum Tower Hospital, Nanjing University, Nanjing, 210008 China; 4 Department of Health Technology and Informatics, Hong Kong Polytechnic University, Hong Kong, 999077 China; 5 Department of Endocrinology, Medical School, The Affiliated Drum Tower Hospital, Nanjing University, Nanjing, 210093 China; 6 Department of Obstetrics and Gynecology, First Affiliated Hospital, Heilongjiang University of Chinese Medicine, Harbin, 150040 China; 7 Nanjing University (Suzhou) High-Tech Institute, Suzhou, 215123 China

**Keywords:** PCOS, Exercise, Irisin, Follicular dysfunction, IRE1α-TXNIP/ROS-NLRP3

## Abstract

**Background:**

Excessive production of androgen drives oxidative stress (OS) and inflammasome activation in ovarian granulosa cells (GCs). Therefore, the induced follicular developmental disorder is the major cause of infertility in women with polycystic ovary syndrome (PCOS). Exercise-induced upregulation of irisin is capable of regulating metabolism by reducing OS and inflammation. Exercise has been shown to alleviate a range of PCOS symptoms, including maintaining a normal menstrual cycle, in several clinical trials.

**Methods:**

Female Sprague–Dawley (SD) rats and primary ovarian cells were treated with two different androgens, dehydroepiandrosterone (DHEA) and dihydrotestosterone (DHT), to simulate a hyperandrogenic environment, followed by eight weeks of exercise training and irisin intervention. The levels of reactive oxygen species (ROS), tissue inflammation and fibrosis were examined using hematoxylin and eosin (H&E) staining, western blot, quantitative real-time PCR (qRT-PCR), dichlorofluorescein diacetate (DCF-DA) probe detection, immunofluorescence staining, immunohistochemistry, and Sirius red staining.

**Results:**

Exercise for eight weeks improved polycystic ovarian morphology and decreased the levels of inflammation, OS, and fibrosis in PCOS rats. Hyperandrogen increased ROS production in ovarian cells by inducing endoplasmic reticulum stress (ERS) and activating the inositol-requiring enzyme 1α (IRE1α)-thioredoxin-interacting protein (TXNIP)/ROS-NOD-like receptor family pyrin domain containing 3 (NLRP3) signaling pathway, further enhancing the levels of inflammation. Irisin suppressed the expression of IRE1α and its downstream targets, thus improving the ovarian dysfunction of PCOS rats induced by hyperandrogen.

**Conclusion:**

Exercise can alleviate various phenotypes of PCOS rats induced by DHEA, and its therapeutic effect may be mediated by secreting beneficial myokines. IRE1α may be an important target of irisin for reducing OS and inflammation, thereby improving ovarian fibrosis.

**Supplementary Information:**

The online version contains supplementary material available at 10.1186/s13048-023-01242-x.

## Introduction

Exercise training is an effective way for improving physical and mental health by enhancing cardiopulmonary function, improving metabolic conditions, maintaining body shape, preventing osteoporosis and boosting immunity. Scientifically rational exercise training as a means for the treatment of chronic diseases has gained international acceptance. Polycystic ovary syndrome (PCOS) is a common reproductive disorder in women of childbearing age worldwide with aetiology arising from the interplay of genetic, epigenetic, lifestyle, and environmental factors. Previous studies have shown that both obese and nonobese PCOS patients show unhealthy lifestyle behaviors, accompanied by a certain degree of aberrant body fat distribution [1], which suggests the importance of lifestyle intervention to disease outcomes in PCOS. According to internationally accepted evidence-based guidelines for the assessment and management of PCOS, lifestyle changes are preferred first-line therapies for patients with PCOS [2]. Lifestyle changes include diet, exercise, behavioral strategies and other comprehensive interventions [3]. Exercise is easier to quantify and unify than dietary modification. The current study shows that exercise can slow the progression of chronic disorders. Physical exercise can improve menstrual irregularities, hirsutism, acnes, hyperandrogenism, and insulin resistance in PCOS, as well as enhance spontaneous and induced ovulation rates, thus contributing to the treatment of infertility in several clinical studies [4–9]. Exercise for eight weeks or even longer periods can decrease the risk of PCOS, including improving BMI, cardiovascular health, anti-Mullerian hormone production, lipid metastasis, hyperandrogenism, oxidative stress (OS), and insulin sensitivity in women with PCOS [10–14]. However, the specific molecular mechanisms accounting for the effectiveness of exercise remain to be further clarified.

The ovarian follicle contains an oocyte, which is surrounded by cumulus cells. The follicular wall is composed of granulosa cells (GCs) and theca cells (TCs). It has been thought that follicular dysfunction induced by hyperandrogen underlies the pathophysiology of PCOS, follicular dysfunction specifically manifested in two aspects: The morphological abnormality of the increase in preantral and antral follicles and the dysfunction of anovulation caused by the lack of dominant follicles. The growth and differentiation of GCs are the key to the initiation and growth of primordial follicles. The functional maturity of GCs is a sign of follicular development. At the later developmental stage of preantral follicles, GCs convert androgen to estrogen via the action of the enzyme aromatase, promoting follicle development and dominant follicle formation. Multiple lines of evidence point to the dysfunction of GCs as a key mechanism for abnormal follicle development. Therefore, identifying key factors that influence GC function may have important implications for prevention and intervention. We have previously reported that PCOS ovaries induced by hyperandrogen demonstrate obvious fibrosis centered on the follicle, which may potentially impact the function of GCs and thus affect the development of follicles [15, 16]. We further discovered that ovarian fibrosis associated with PCOS is also related to hyperandrogen-induced excessive OS and NOD-like receptor family pyrin domain containing 3 (NLRP3) inflammasome activation [17, 18].

Under physiological and pathophysiological conditions, perturbations in endoplasmic reticulum (ER) homeostasis lead to an unfolded protein response (UPR) and endoplasmic reticulum stress (ERS). Inositol-requiring enzyme 1α (IRE1α) is involved in maintaining ER homeostasis. When separated from ER chaperone-binding immunoglobulin protein (BIP), IRE1α initiates UPR signaling and induces OS, inflammation, and cell death [19, 20]. Recent studies have demonstrated that ERS can facilitate the assembly and activation of the NLRP3 inflammasome. Thioredoxin-interacting protein (TXNIP) is an important protein linking OS to inflammation. ERS induces reactive oxygen species (ROS) production, and TXNIP detaches from thioredoxin (TRX) to associate with the NLRP3 inflammasome, resulting in NLRP3 inflammasome activation [21].

Skeletal muscles make up 40% of total body weight and play important roles in physical activity across the life course. In recent years, accumulating evidence has shown that skeletal muscle is not only recognized as a motor organ but also an endocrine organ which has a powerful endocrine function. Skeletal muscles can regulate glucose and lipid metabolism in autocrine, paracrine, or endocrine ways or influence the metabolism and functions of other organs and tissues by endocrine mechanisms. This is an important mechanism that mediates exercise adaptation. Investigators defined the myogenic secretory factor as ‘myokines’ [22, 23]. Some of the myokines can reportedly regulate metabolism, alleviate disease severity and retard disease progression. Exercise-induced myokines may play a pivotal role in PCOS phenotype improvement, and their precise mechanism of action remains to be thoroughly studied. Myokines include insulin-like growth factor-I (IGF-1), fibroblast growth factor-2 (FGF-2), myostatin, irisin, myonectin, interleukin-6 (IL-6), IL-7, IL-15, bone morphogenetic protein (BMP), osteoglycin (OGN), as well as many other secretory factors [24]. Fibronectin type III domain-containing protein-5 (FNDC5) is cleaved at the C-terminus to give rise to irisin, a myokine that has been recently discovered, as a result of peroxisome proliferator-activated receptor-γ coactivator-1α (PGC-1α) activation during exercise [25, 26]. The most prominent role of irisin is the conversion of white adipose tissue to brown adipose tissue, thereby increasing whole-body energy expenditure [27, 28]. In recent years, an increasing number of studies have also shown that irisin can play an important anti-inflammatory role [29], thereby reducing fibrosis [30, 31] and disease severity [32–34]. Prompted by these investigations, our main hypothesis was that the mechanism by which exercise improves PCOS is through the secretion of beneficial myokines. Irisin may play an important role in inhibiting OS and inflammation via the IRE1α-TXNIP/ROS-NLRP3 signaling pathway.

## Materials and methods

### Animals and experiment protocol

Wild-type female Sprague–Dawley (SD) rats (21 days old, 50–60 g, *n* = 26) were obtained from Junke Biotechnology Corporation, China. The rats were maintained in a specific pathogen-free (SPF) environment (Jiangsu Key Laboratory of Molecular Medicine) with a 12-h light/dark cycle at 24 ± 1 °C. Enough food and water were provided for free access.

At postnatal day 23, rats of comparable body weights were randomly divided into three experimental groups (oil, *n* = 8; dehydroepiandrosterone (DHEA, Sigma, USA), *n* = 9; DHEA + exercise (D + E), *n* = 9). To create a PCOS model, rats in the DHEA and D + E groups received a daily hypodermic injection of DHEA (6 mg/100 (g·d)) for 35 consecutive days [35]. The oil group rats (*n* = 8), which were used as controls, received a daily hypodermic injection of an equal volume of experimental grade soybean oil purchased from Yuanye Biological Technology Corporation, China.

After the modeling was completed, three rats from the oil and DHEA groups were killed, and their bilateral ovaries, blood, and various other tissues were harvested for tissue sectioning and subsequent molecular experiments. At the same time, the rats in the D + E group were treated with flat treadmill exercise (Sansbio, China) intervention for eight weeks (1 h (5 m/minute, 5 min; 10 m/minute, 10 min; 20 m/minute, 45 min)/day, 6 days/week). Rats in the oil and DHEA groups were left untreated.

On day 92, all rats were killed, and both ovaries were harvested. Next, we removed fat around the ovary. Blood and various other tissues were harvested, and immediately stored at -80 °C for tissue sectioning and molecular analysis. The experiments were carried out following the principles and guidelines for the use of laboratory animals and were approved by the institutional research animal committee of Nanjing University.

### Isolation and culture of GCs and TCs

Female rats were injected with pregnant mare serum gonadotropin (PMSG, Sansheng Biological Technology Corporation, China) (20 IU) to promote the development of multiple follicles. Forty-eight hours after the injection, the rats were killed by anesthesia with 0.3% sodium pentobarbital, and the ovaries were isolated. Next, the follicles from the ovary were peeled with micro tweezers. The follicle was punctured to release GCs, and a 70-μm cell strainer was used to remove cell debris.

The follicles that released GCs were collected and digested in 5 mL DMEM-F12 (Gibco, USA) medium containing 0.35 mg/mL collagenase IV (Sigma), 10 μg/mL DNase I (PanReac AppliChem, Germany), and 10 mg/mL bovine serum albumin (BSA, BioSharp, China) at 37 °C for 30 min. After digestion, the samples were centrifuged at 1000 rpm for 5 min and the supernatant was discarded. The TCs were resuspended in 5 mL of fresh DMEM-F12. Cell debris was removed by a 100-μm cell strainer.

Primary GCs and TCs were cultured in DMEM-F12 containing 10% fetal bovine serum (FBS, Gibco) and 1% penicillin–streptomycin solution (Gibco) at 37 °C with 5% CO_2_. GCs and TCs were treated with various concentrations of dihydrotestosterone (DHT, Meilun Biological Technology Corporation, China) as indicated and irisin (10 ng/mL). In addition, the protein and mRNA levels of various factors in GCs and TCs that are possibly regulated by DHT or irisin were analyzed. Furthermore, GCs and TCs were treated with small interfering RNA (siRNA) in the presence of 5 μM DHT. Next, GCs and TCs were lysed for further analysis.

### IRE1α knockdown by siRNA and siRNA-containing lentiviral vectors

The IRE1α siRNA target sequence (5’-AUGACGUGGACUACAAGAUGUTT-3’) and the control siRNA sequence (5’-UUCUCCGAACGUGUCACGUdTdT-3’) were designed at Keygen Biotech (Keygen Biotech, China). Primary GCs and TCs were transfected with the siRNA using Lipofectamine 2000 (Invitrogen, USA) with a 72-h incubation. Next, the cells were treated with DHT (5 μM for 24 h) for various assays.

### Serum hormone measurement

Blood samples were collected from the superior vena cava of rats and stored at -80 °C followed by immediate centrifugation. Next, luteinizing hormone (LH), follicle stimulating hormone (FSH), and irisin levels were analyzed by enzyme-linked immunosorbent assay (ELISA, Elabscience Biotechnology, China).

### Hematoxylin and eosin (H&E) staining

Ovarian and abdominal adipose tissues were fixed, embedded in paraffin, and processed on slides for H&E staining to examine the histological changes of the ovary and abdominal adipose.

### Immunohistochemistry

Samples of ovarian tissues were sectioned at a thickness of 4 μm and stained with specific antibodies against IRE1α (1:100, Proteintech, China). Next, the sections were incubated with a secondary goat anti-rabbit IgG (H + L) HRP. Images were captured using an optical microscope (Leica Microsystems, Germany).

### Masson staining and Sirius red staining

Ovary sections were stained with Masson staining and Sirius red to visualize collagen deposition.

### Immunofluorescence

Tissue sections were blocked with 3% BSA for 30 min at 25 °C. Sections were incubated overnight at 4 °C with antibodies against integrin αVβ5 (1:100, Santa Cruz Biotechnology, Japan). For GC and TC staining, sections were fixed in 4% paraformaldehyde (Servicebio, China) for 30 min at 25 °C and then permeabilized with 0.3% Triton X-100 (Beyotime, China). After washing with PBS three times, the cells were blocked with 3% BSA for 30 min at 25 °C. Cells were incubated with antibodies against IRE1α (1:100, Proteintech), apoptosis-associated speck-like protein containing a CARD (ASC, 1:100, AdipoGen Life Science, USA), NLRP3 (1:100, CST, USA), a-smooth muscle actin (α-SMA, 1:100, Abcam, UK) and collagen I (1:100, Bioworld Technology, China) overnight at 4 °C. After washing with PBS three times, tissue sections and cells were incubated at 25 °C for 2 h with fluorescent secondary antibodies (Beyotime). Nuclei were counterstained with 4’,6-diamidino-2-phenylindole (DAPI, Beyotime) at a dilution of 1:2000 for 30 min and photographed using an Olympus laser scanning confocal microscope (FV3000, Japan).

### Measurement of intracellular ROS production

To stain intracellular ROS, cells were plated on glass-bottomed 24-well plates and were incubated with dichlorofluorescein diacetate (DCF-DA, MCE, USA) (10 μM) for 30 min at 37 °C following DHT and siRNA treatment. The medium was discarded and the cells were gently washed three times with PBS. The images of the cells were captured using an Olympus laser scanning confocal microscope (FV3000).

### Measurement of malondialdehyde (MDA) and superoxide dismutase (SOD) levels

MDA and SOD were measured to assess the OS level. The MDA and SOD levels in the serum, GCs and TCs were measured using the Lipid Peroxidation MDA Assay Kit (Beyotime) and SOD Activity Assay Kit (Beyotime).

### Cell Counting Kit-8 (CCK8) analysis

Primary GCs and TCs were seeded in 96-well plates (1 × 10^5^ cells/well) and cultured for 48 h. Next, the cells were treated with various concentrations of irisin (0, 1, 2, 5, 10, 20 ng/mL) pretreatment for 6 h and then DHT (5 μM) treatment for 48 h. Cell viability was measured by CCK8 (MCE). CCK8 was added to the plates and incubated for 3 h. The absorbance was determined by a microplate reader at 450 nm. The experiment was repeated three times to obtain the mean values.

### Western blot

Ovary, GC and TC lysates were extracted by RIPA lysis buffer (Beyotime) containing 1 mM Pierce™ Phosphatase Inhibitor (Selleck, USA) and 0.1% Halt™ Protease Inhibitor Cocktail (Selleck). Equal amounts of total proteins were separated by 10% sodium dodecyl sulfate–polyacrylamide gel electrophoresis, and the protein bands were then transferred onto polyvinylidene difluoride membranes (Merck Millipore, USA). Target bands were incubated with corresponding primary antibodies against IRE1α (1:1000, Proteintech), NLRP3 (1:1000, CST), ASC (1:1000, AdipoGen Life Science), gasdermin D (GSDMD, 1:1000, Proteintech), gasdermin E (GSDME, 1:1000, Abcam), C-terminal fragment of gasdermin D (GSDMD-C, 1:1000, Abcam), interleukin-1β (IL-1β, 1:1000, Abcam), interleukin-18 (IL-18, 1:1000, Abnova, USA), androgen receptor (AR, 1:1000, Abcam), cytochrome p450 11 (CYP11α1, 1:1000, Bioworld Technology), cytochrome p450 19 (CYP19α1, 1:1000, Bioworld Technology), transforming growth factor-beta (TGF-β, 1:1000, CST), α-SMA (1:1000, Abcam), P-SMAD3 (1:1000, CST), β-catenin (1:1000, CST), collagen I (1:1000, Bioworld Technology) and glyceraldehyde-3-phosphate dehydrogenase (GAPDH, 1:5000, Bioworld Technology) overnight at 4 °C, followed by the addition of HRP-labeled secondary antibodies (1:40,000, Bioworld Technology). The blots were visualized using chemiluminescent detection (Merck Millipore). Densitometric analysis was performed with ImageJ.

### Quantitative real-time PCR (qRT-PCR)

Total RNA from tissues and cells was extracted by using TRIzol reagent (Beyotime), and cDNA was synthesized with a reverse transcription kit (Vazyme Biotech, China). QRT-PCR was performed with the ABI Viia 7 Real-Time PCR system (ABI, USA) by using SYBR Green PCR Master Mix (Vazyme Biotech), and the primers are shown in Table 1. The critical threshold cycle (Ct) value was determined for each reaction, which was transformed into relative quantification data using the 2^−ΔΔCt^ method. The housekeeping gene β-actin was used as an internal control.

**Table 1:** Primers of the genes used in the study

Genes	Forward	Reverse
β-actin	5’-TTCCTTCCTGGGTATGGAAT-3’	5’-GAGGAGCAATGATCTTGATC-3’
TGF-β	5’-TACTGCTTCAGCTCCACAGAGA-3’	5’-CAGACAGAAGTTGGCATGGTAG-3’
α-SMA	5’-AGGGACTAATGGTTGGAATGG-3’	5’-CAATCTCACGCTCACGCTCGGCAGTAG-3’
β-catenin	5’-ACCATCGAGAGGGCTTGTTG-3’	5’-CGCACTGCCATTTTAGCTCC-3’
Fibronectin	5’-TGACAACTGCCGTAGACCTGG-3’	5’-TACTGGTTGTAGGTGTGGCCG-3’
NLRP3	5’-CAGCGATCAACAGGCGAGAC-3’	5’-AGAGATATCCCAGCAAACCTATCCA-3’
ASC	5’-GGACCAACACAGGCAAGCACTC-3’	5’-ACAAGTTCTTGCAGGTCAGGTTCC-3’
IL-1β	5’-CTACCTATGTCTTGCCCGTGGAG-3’	5’-GGGAACATCACACACTAGCAGGTC-3’
TXNIP	5’-AGATAGAGTATATCTTCAAGCCG-3’	5’-CTATGTGCTGGCTTTGGT-3’
GSDMD	5’-TTGAGTGTCTGGTGCTCGAC-5’	5’-ATGGGGTGCTCTGTTCCAAG-5’
IRE1α	5’-CCAACCACTCACTCAACTCT-3’	5’-TTTTCCCAACAATCACCA-3’
PGC-1α	5’-ACATCGCAATTCTCCCTT-3’	5’-CTCTTGAGCCTTTCGTGCTC-3’
FNDC5	5’-TGGAGGAGGACACAGAGTATATCG-3’	5’-CATATCTTGCTTCGGAGGAGACC-3’
IL-6	5’-TATGAACAGCGATGATGCACTG-3’	5’-TTGCTCTGAATGACTCTGGCTT-3’
IL-15	5’-GACAGTGACTTTCATCCCAGTT-3’	5’-CATTCCTTGCAGCCAGAC-3’
Angptl4	5’-AGAAGTTGGAGATGCAGAGGGAC-3’	5’-CCACAAGAGCACCATTGAGTGTAT-3’
FGF-2	5’-CAGTGAGTGCCGACCCGCTC-3’	5’-GCGGGAAGACAGCCAGTCCG-3’
Myostatin	5’-ATCTGAGAGCCGTCAAGACTCC-3’	5’-CAGTCAAGCCCAAAGTCTCTCC-3’
αVβ5	5’-TGCCAAGTTCCAAAGCG-3’	5’-GGTCCAAGGAGTCCGAGAC-3’

### Statistics

Statistical analyses were performed by GraphPad Prism 7.00 software. A two-tailed unpaired Student’s t test was used for comparing two groups. One-way analysis of variance (ANOVA) was used for comparing more than two groups, followed by the Bonferroni post hoc test. The Kruskal–Wallis test was performed for the comparisons of data with nonnormal distribution or heterogeneity of variance. The quantitative data are shown as the means ± standard error of the mean (SEM). A *P* value ≤ 0.05 was considered statistically significant.

## Results

### Exposure to hyperandrogen drives ovarian dysfunction by ERS in PCOS rats

**Fig. 1:**
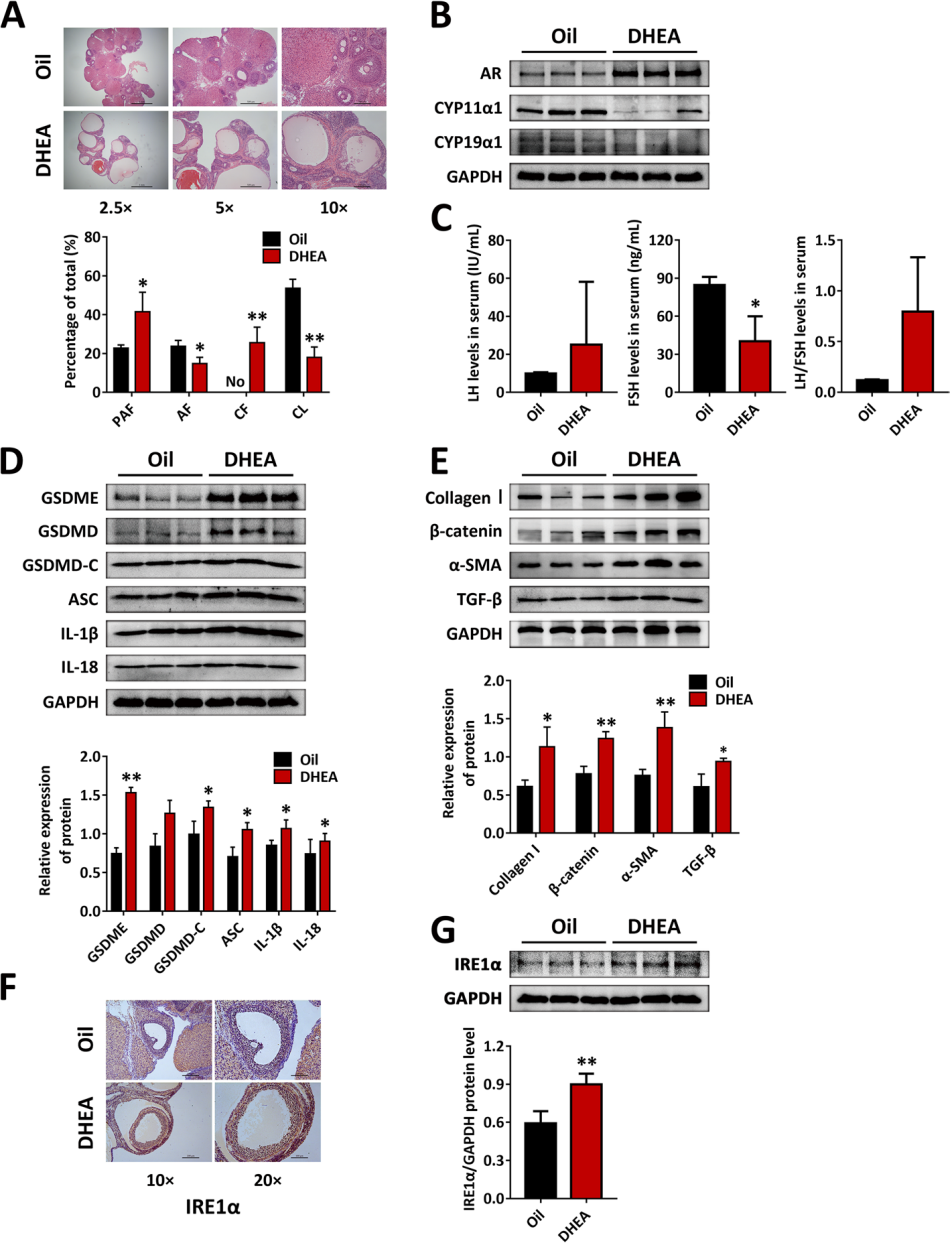
DHEA induces ovarian dysfunction, and IRE1α levels are increased in the ovaries of PCOS rats. Rats received DHEA for induction of polycystic ovarian syndrome. **A** H&E staining was carried out for showing ovarian and follicular morphology (upper panel). The percentages of each type of the follicles were quantified (lower panel). **B** The protein levels of AR and cytochrome P450 family enzymes (CYP11α1, CYP19α1) in ovaries were assessed by western blot. **C** Serum LH and FSH levels were analyzed using ELISA. **D-E** The expression of NLRP3 inflammasome activation factors (GSDME, GSDMD, GSDMD-C, ASC, IL-1β and IL-18) (**D**) and fibrosis factors (collagen I, β-catenin, α-SMA, TGF-β) (**E**) in ovaries was assessed by western blot. **F** IRE1α expression in ovaries was analyzed by immunohistochemical staining. **G** Levels of IRE1α were measured using western blot. Data are shown as the mean ± SD. *p ≤ 0.05, **p ≤ 0.01. DHEA, dehydroepiandrosterone; AR, androgen receptor; PAF, preantral and early antral follicle; AF, antral follicle; CF, cystic follicles; CL, corpus luteum; CYP11α1, cytochrome p450 11; CYP19α1, cytochrome p450 19; GAPDH, glyceraldehyde-3-phosphate dehydrogenase; LH, luteinizing hormone; FSH, follicle stimulating hormone; GSDME, gasdermin E; GSDMD, gasdermin D; GSDMD-C, C-terminal fragment of gasdermin D; ASC, apoptosis-associated speck-like protein containing a CARD; IL-1β, interleukin-1β; IL-18, interleukin-18; α-SMA, a-smooth muscle actin; TGF-β, transforming growth factor-beta; IRE1α, inositol-requiring enzyme 1α

In this study, we observed that the ovaries in DHEA-treated rats had dramatically more preantral, early antral and cystic follicles but almost no corpus luteum. The number and layers of GCs in the cystic follicles were decreased (Fig. 1A). In addition, the ovarian protein levels of AR were profoundly enhanced in DHEA-treated rats. CYP11α1 is the first rate-limiting enzyme in the production of steroid hormones, and CYP19α1 participates in the conversion of androgen to estrogen. Our data revealed that CYP11α1 and CYP19α1 were markedly downregulated in DHEA-treated rats (Fig. 1B). Next, serum sex hormone levels were measured in the two groups of rats. In DHEA-treated rats, the LH level was increased, the FSH level was markedly decreased, and the LH/FSH level demonstrated a trend of enhancement compared with the normal control rats (Fig. 1C). Our data support the successful construction of a PCOS rat model.

Furthermore, the inflammation and fibrosis levels of ovaries were measured. The protein levels of NLRP3 inflammasome activation factors, such as GSDME, GSDMD, GSDMD-C, ASC, IL-1β, and IL-18 (Fig. 1D), fibrosis factors, such as collagen I, β-catenin, α-SMA, and TGF-β, were markedly higher in the ovaries of DHEA-treated rats (Fig. 1E), which indicates that excessive DHEA was able to induce ovarian dysfunction in rats. Next, we attempted to identify the upstream mechanism by which hyperandrogens induce ovarian inflammation and fibrosis. IRE1α is an ER transmembrane sensor that is activated when the cell undergoes a UPR. We found that DHEA treatment promoted the expression of IRE1α in GCs in antral follicles (Fig. 1F), and the protein level of IRE1α in the ovarian tissue of DHEA-treated rats was significantly increased (Fig. 1G). This indicates that the activation of IRE1α may take part in hyperandrogenism-induced ovarian dysfunction.

### IRE1α is a key molecule in DHT-induced dysfunction of GCs

**Fig. 2:**
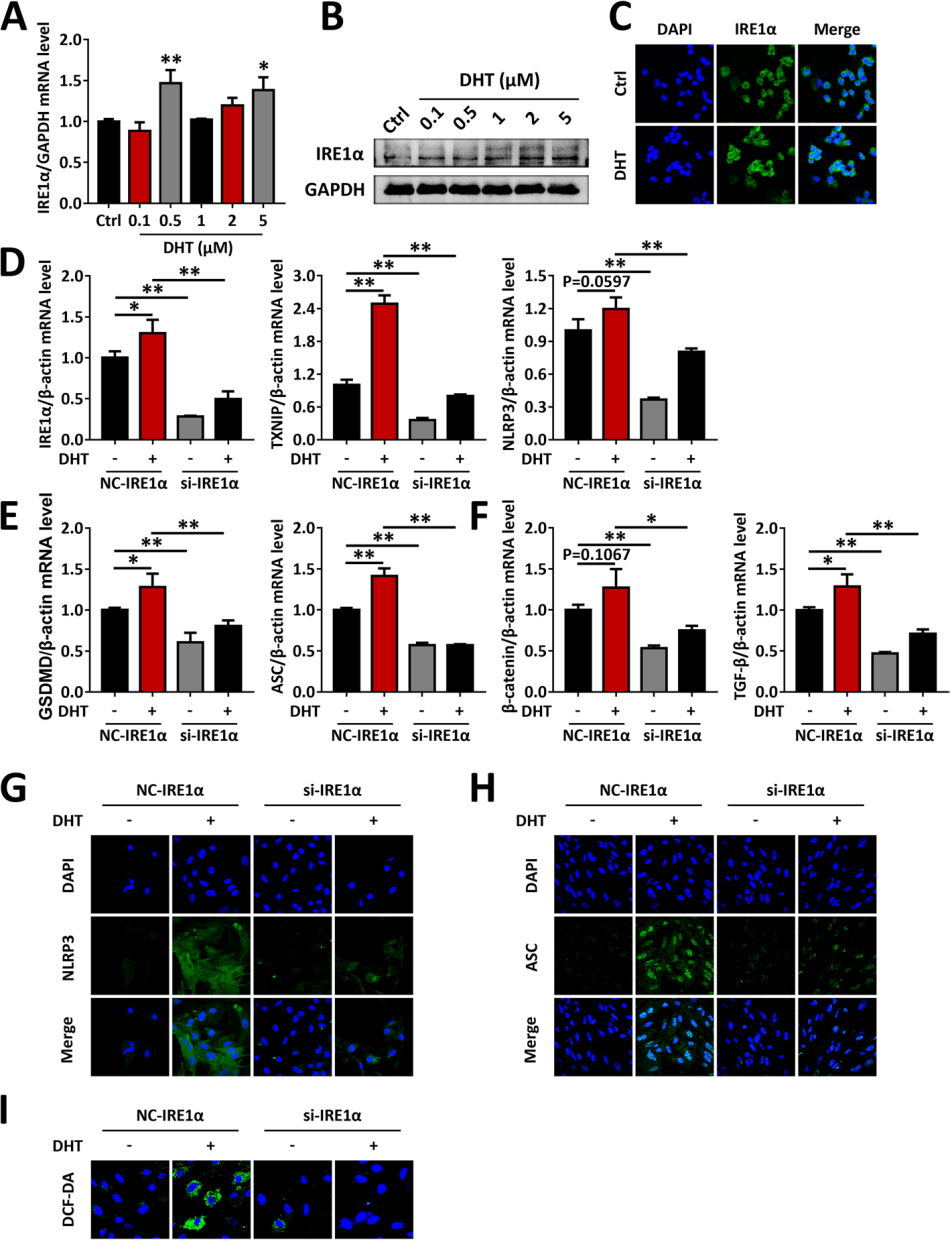
IRE1α silencing by siRNA rescues DHT-induced dysfunction of GCs. GCs from naïve rats were treated with DHT, or were transfected with IRE1α siRNA followed by treatment with DHT. **A** The mRNA levels of IRE1α in GCs were analyzed by qRT-PCR. **B** The protein levels of IRE1α in GCs were analyzed by western blot. **C** Levels of IRE1α in GCs (Alexa Fluor 488) were analyzed by immunofluorescence staining using an Alexa Fluor 488-conjugated antibody (60 ×). **D-F** The expression of IRE1α, TXNIP, NLRP3 (**D**), NLRP3 inflammasome activation factors (GSDMD, ASC) (**E**) and fibrosis factors (β-catenin, TGF-β) (**F**) in GCs were assessed by qRT-PCR. **G-H** The protein levels of ASC (**G**) and α-SMA (**H**) in GCs were analyzed by immunofluorescence staining (60 ×). **I** ROS in GCs were assessed by the DCF-DA probe using confocal microscopy (90 ×). Data are shown as the mean ± SD. *p ≤ 0.05, **p ≤ 0.01. IRE1α, inositol-requiring enzyme 1α; GAPDH, glyceraldehyde-3-phosphate dehydrogenase; Ctrl, control; DHT, dihydrotestosterone; DAPI, 4’,6-diamidino-2-phenylindole; NC, normal contrast; si, siRNA; TXNIP, thioredoxin-interacting protein; NLRP3, NOD-like receptor family pyrin domain containing 3; GSDMD, gasdermin D; ASC, apoptosis-associated speck-like protein containing a CARD; TGF-β, transforming growth factor-beta; DCF-DA, dichlorofluorescein diacetate; ROS, reactive oxygen species

Consistently, in vitro, DHT exposure increased the protein and mRNA levels of IRE1α in rat primary ovarian GCs (Fig. 2A-B), IRE1α was mainly expressed in the cytoplasm, and DHT treatment markedly elevated the protein level of IRE1α, as confirmed by immunofluorescence staining (Fig. 2C). To determine whether IRE1α is a key molecule in the dysfunction of ovarian GCs induced by DHT, rat primary GCs were transfected with siRNA to knockdown IRE1α. TXNIP is a key molecule that links OS with the activation of inflammasomes. The qRT-PCR data showed that IRE1α knockdown inhibited the DHT-induced enhancement of TXNIP and NLRP3 in GCs (Fig. 2D). Furthermore, NLRP3 inflammasome activation-related factors (GSDMD, ASC) and fibrosis-related factors (β-catenin, TGF-β) were eliminated by IRE1α silencing (Fig. 2E-F). Immunofluorescence staining also confirmed similar results: si-IRE1α markedly rescued the expression of NLRP3 and ASC in GCs induced by DHT (Fig. 2G-H). In addition, treatment with si-IRE1α in GCs inhibited androgen-induced ROS accumulation according to the DCF-DA probe analysis (Fig. 2I). Similar results were obtained in TCs (Supplementary Fig. 2). Taken together, our findings suggest that IRE1α silencing by siRNA ameliorates DHT-induced dysfunction of GC and TC.

### Exercise suppresses the IRE1α-TXNIP/ROS-NLRP3 pathway and improves the phenotypes of PCOS rats

**Fig. 3:**
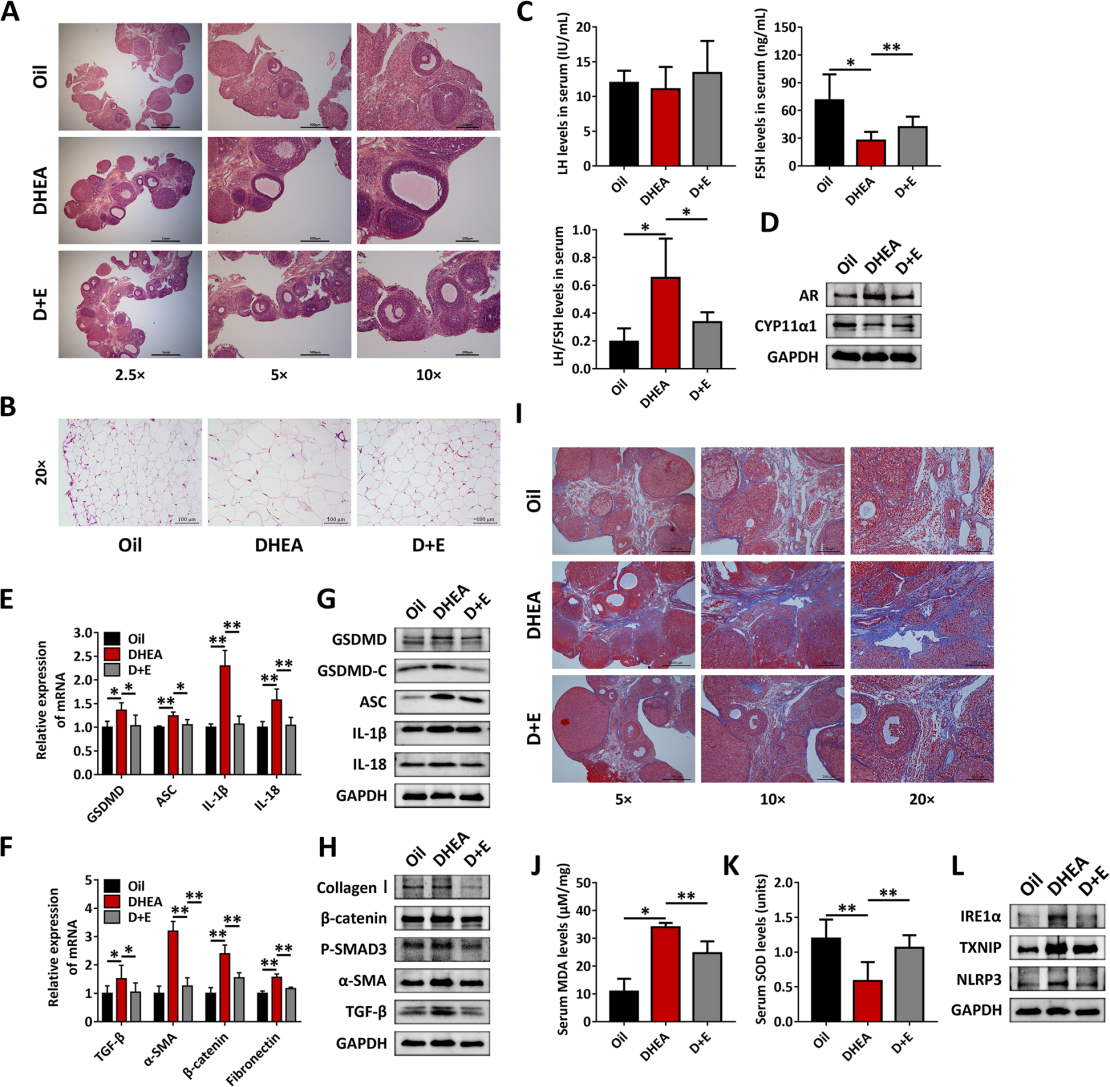
Exercise suppresses the IRE1α-TXNIP/ROS-NLRP3 pathway and improves the phenotype of PCOS rats. Rats received DHEA to induce polycystic ovary syndrome followed by exercise treatment. **A** Tissues were stained with H&E for revealing ovarian and follicular morphology. **B** Abdominal adipose tissue were stained with H&E. **C** Serum LH and FSH levels were analyzed using ELISA. **D** The expression of AR and CYP11α1 in ovaries was assessed by western blot. **E–F** The expression of NLRP3 inflammasome activation factors (GSDMD, ASC, IL-1β and IL-18) (**E**) and fibrosis factors (TGF-β, α-SMA, β-catenin and fibronectin) (**F**) in ovaries was assessed by qRT-PCR. (**G-H**) The expression of NLRP3 inflammasome activation factors (GSDMD, GSDMD-C, ASC, IL-1β and IL-18) (**G**) and fibrosis factors (collagen I, β-catenin, P-SMAD3, α-SMA, TGF-β) (**H**) in ovaries was assessed by western blot. **I** Collagen in ovarian slices was revealed by Masson staining. **J-K** Serum MDA levels (**J**) and SOD activity (**K**) were analyzed using an enzymatic colorimetric method. **L** The expression levels of IRE1α, TXNIP and NLRP3 in ovaries were analyzed by western blot assay. Data are shown as the mean ± SD. *p ≤ 0.05, **p ≤ 0.01. DHEA, dehydroepiandrosterone; D + E, DHEA + Exercise; LH, luteinizing hormone; FSH, follicle stimulating hormone; AR, androgen receptor; CYP11α1, cytochrome p450 11; GAPDH, glyceraldehyde-3-phosphate dehydrogenase; GSDMD, gasdermin D; ASC, apoptosis-associated speck-like protein containing a CARD; IL-1β, interleukin-1β; IL-18, interleukin-18; TGF-β, transforming growth factor-beta; α-SMA, a-smooth muscle actin; GSDMD-C, C-terminal fragment of gasdermin D; MDA, malondialdehyde; SOD, superoxide dismutase; IRE1α, inositol-requiring enzyme 1α; TXNIP, thioredoxin-interacting protein; NLRP3, NOD-like receptor family pyrin domain containing 3

Furthermore, we performed flat treadmill exercise intervention on DHEA-treated rats for eight consecutive weeks. The ovarian morphology of DHEA-treated rats recovered partially, but compared with the exercise group, there were still more atypical follicles in the ovaries of nonexercised rats (Fig. 3A). Exercise treatment resulted in significantly smaller lipid droplets in abdominal fat in the group compared with DHEA treatment group (Fig. 3B). In addition, exercise elevated FSH levels and markedly reduced LH/FSH levels (Fig. 3C). After exercise, the AR protein level in the ovarian tissue of DHEA-treated rats was decreased, and the CYP11α1 protein level was increased (Fig. 3D). The above results show that eight weeks of aerobic exercise improved the ovarian morphology, sex hormone levels and ovarian function of DHEA-treated rats to a certain extent. Simultaneously, at the mRNA and protein expression levels, the degree of NLRP3 inflammasome activation and fibrosis in the ovaries of DHEA-treated rats was reduced after exercise (Fig. 3E-H). Masson staining and Sirius red staining revealed collagen deposition in the ovarian stroma of DHEA-treated rats, which disappeared after exercise (Fig. 3I and Supplementary Fig. 2).

Furthermore, we attempted to explore the specific mechanism by which exercise improves PCOS and whether it is related to the inhibition of activated IRE1α. Exercise reduced the serum MDA level and increased the serum SOD level in DHEA-treated rats (Fig. 3J-K). Furthermore, the experimental results showed that the expression of IRE1α, TXNIP and NLRP3 in the ovarian tissue of DHEA-treated rats was decreased profoundly after exercise (Fig. 3L). This result indicated that the improvement of the PCOS phenotype by exercise is related to the inhibition of the IRE1α-TXNIP/ROS-NLRP3 signaling pathway.

### Exercise and myokine induction inhibit ROS production and improve follicular development in PCOS rats

**Fig. 4:**
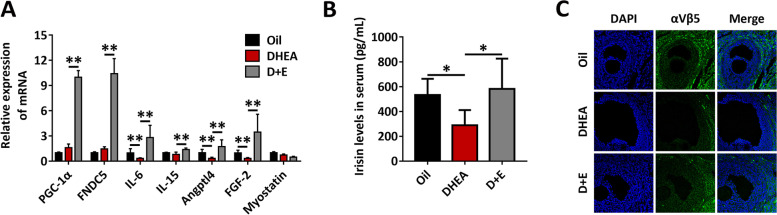
Exercise and myokine induction inhibit ROS production and improve follicular development in PCOS rats. Rats received DHEA to induce polycystic ovary syndrome followed by exercise treatment. **A** The RNA levels of PGC-1α, FNDC5, IL-6, IL-15, Angptl4, FGF-2, and myostatin were analyzed by qRT-PCR. **B** Serum irisin levels were analyzed using ELISA. **C** The expression of integrin αVβ5 in ovaries was analyzed by immunofluorescence staining (50 ×). Data are shown as the mean ± SD. *p ≤ 0.05, **p ≤ 0.01. DHEA, dehydroepiandrosterone; D + E, DHEA + Exercise; PGC-1α, peroxisome proliferator-activated receptor-γ coactivator-1α; FNDC5, fibronectin type III domain-containing protein-5; IL-6, interleukin-6; IL-15, interleukin-15; FGF-2, fibroblast growth factor 2; αVβ5, integrin αVβ5

To determine the key beneficial myokines, the mRNA levels of several myokines in rat skeletal muscle were tested. The irisin precursor FNDC5 and its upstream molecule PGC-1α increased by more than tenfold (Fig. 4A). Correspondingly, the serum irisin level in DHEA-treated rats decreased and returned to normal level after exercise (Fig. 4B). These results suggest that exercise to improve PCOS may be mediated by muscle secretion of irisin. The integrin αVβ5 receptor has been shown to be the receptor for irisin [36]. Therefore, we aimed to confirm the existence of integrin αVβ5 receptors on the ovaries of rats. Indeed, the integrin αVβ5 receptor as profoundly expressed in TCs, and the expression level was moderate in GCs through immunofluorescence staining (Fig. 4C).

### Irisin mediates skeletal muscle-ovary crosstalk and inhibits the IRE1α-TXNIP/ROS-NLRP3 signaling pathway

**Fig. 5:**
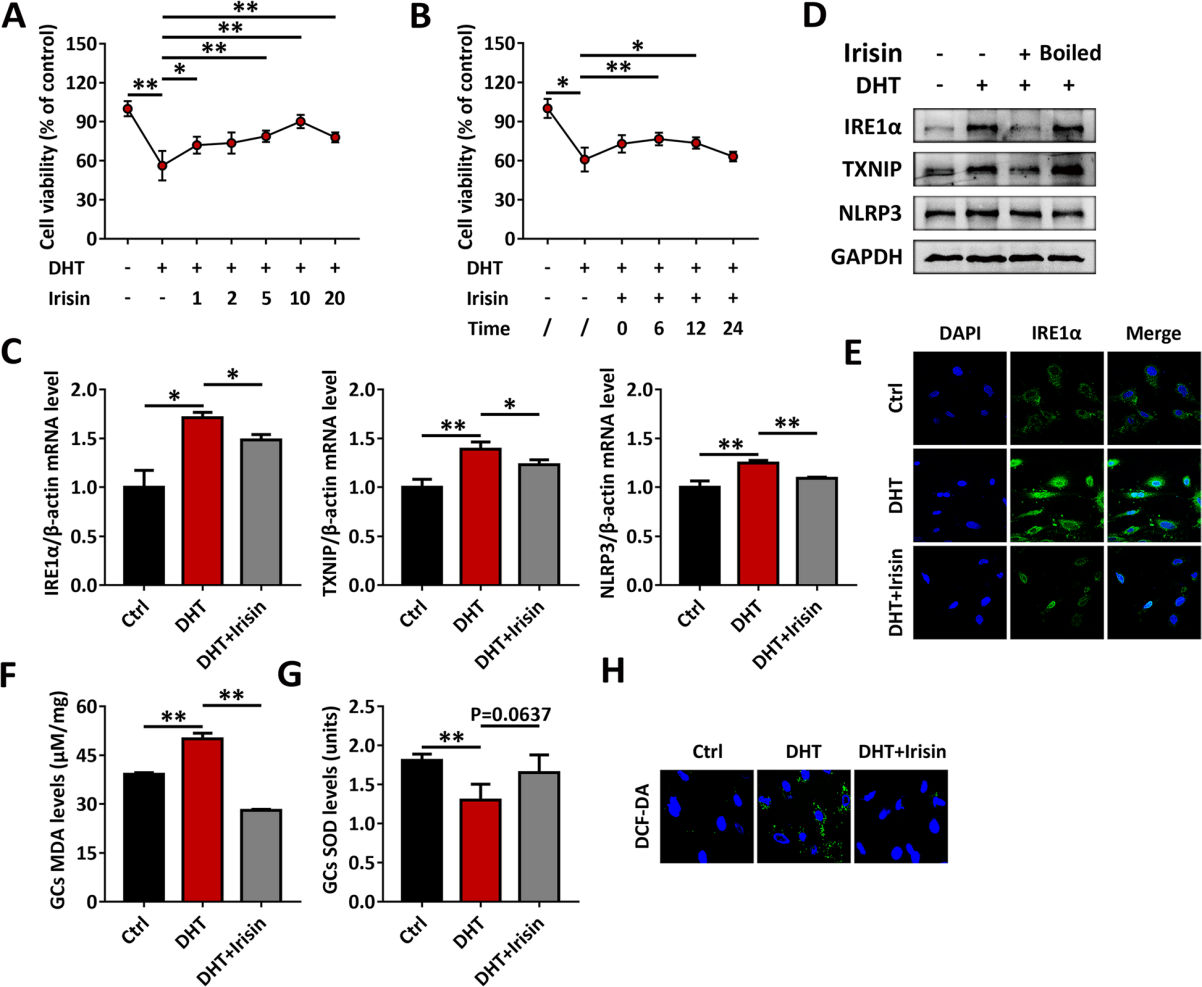
Irisin mediates skeletal muscle-ovary crosstalk and inhibits the IRE1α-TXNIP/ROS-NLRP3 signaling pathway in GCs. GCs were treated with DHT, in the presence or absence of various concentrations of irisin. **A-B** Cell viability was measured by CCK8. (**C-D**) The mRNA (**C**) and protein (**D**) levels of IRE1α, TXNIP and NLRP3 in GCs were analyzed by qRT-PCR and western blot. (**E**) The expression of IRE1α in GCs was analyzed by immunofluorescence staining (60 ×). **F-G** GC MDA levels (**F**) and SOD activity (**G**) were analyzed using an enzymatic colorimetric method. **H** ROS in GCs was assessed by the DCF-DA probe using confocal microscopy (90 ×). Data are shown as the mean ± SD. *p ≤ 0.05, **p ≤ 0.01. DHT, dihydrotestosterone; IRE1α, inositol-requiring enzyme 1α; TXNIP, thioredoxin-interacting protein; NLRP3, NOD-like receptor family pyrin domain containing 3; GAPDH, glyceraldehyde-3-phosphate dehydrogenase; Ctrl, control; DAPI, 4’,6-diamidino-2-phenylindole; MDA, malondialdehyde; SOD, superoxide dismutase; DCF-DA, dichlorofluorescein diacetate; CCK8, Cell Counting Kit-8

Next, we aimed to confirm whether irisin mediates the beneficial effects of exercise. GCs were treated with various concentrations of DHT for 48 h to simulate a PCOS hyperandrogen environment. The cell viability of GCs decreased to less than 60% after 5 μM DHT treatment. Irisin pretreatment significantly increased the viability of GCs under hyperandrogen exposure (Fig. 5A). The peak response appeared at 6 h after treatment of GCs with irisin (Fig. 5B). Furthermore, we wanted to explore the mechanism by which irisin improves the viability of GCs in a hyperandrogen environment. Irisin treatment significantly reduced the mRNA level of IRE1α in GCs, and the mRNA levels of TXNIP and NLRP3 were also significantly inhibited by irisin (Fig. 5C). At the protein level, irisin was also confirmed to have a beneficial effect on GCs: IRE1α, TXNIP and NLRP3 in GCs were markedly decreased at the protein level following treatment with 10 ng/mL irisin, and this effect disappeared when irisin was boiled at 100 °C for 10 min (Fig. 5D). Immunofluorescence staining also confirmed the inhibitory effect of irisin on IRE1α (Fig. 5E). Furthermore, irisin indeed reduced the level of MDA (Fig. 5F) and increased the level of SOD in GCs exposed to DHT (Fig. 5G). ROS in GCs was analyzed using the DCF-DA probe. The results showed that the enhancement of ROS stimulated by DHT was greatly inhibited by irisin pretreatment (Fig. 5H).

**Fig. 6:**
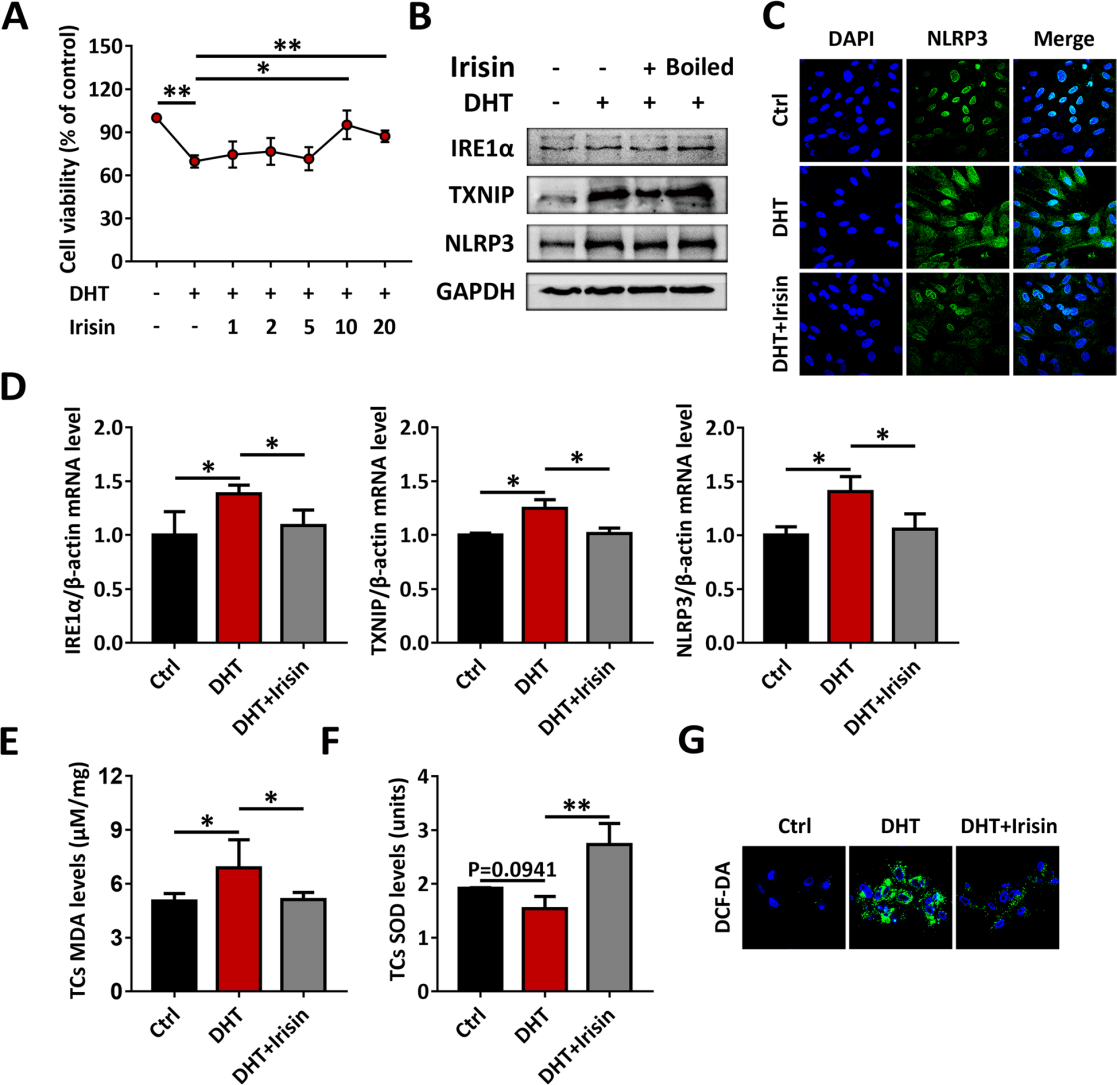
Irisin mediates skeletal muscle-ovary crosstalk and inhibits the IRE1α-TXNIP/ROS-NLRP3 signaling pathway in TCs. TCs were treated with DHT, in the presence or absence various concentrations of irisin. **A** Cell viability was measured by CCK8. **B** and **D** The protein (**B**) and mRNA (**D**) levels of IRE1α, TXNIP and NLRP3 in TCs were analyzed by western blot and qRT-PCR. (**C**) The expression of NLRP3 in TCs was analyzed by immunofluorescence staining (60 ×). **E–F** TC MDA levels (**E**) and SOD activity (**F**) were analyzed using an enzymatic colorimetric method. **G** ROS in TCs was assessed by the DCF-DA probe using confocal microscopy (90 ×). Data are shown as the mean ± SD. *p ≤ 0.05, **p ≤ 0.01. DHT, dihydrotestosterone; IRE1α, inositol-requiring enzyme 1α; TXNIP, thioredoxin-interacting protein; NLRP3, NOD-like receptor family pyrin domain containing 3; GAPDH, glyceraldehyde-3-phosphate dehydrogenase; Ctrl, control; DAPI, 4’,6-diamidino-2-phenylindole; MDA, malondialdehyde; SOD, superoxide dismutase; DCF-DA, dichlorofluorescein diacetate; CCK8, Cell Counting Kit-8

In view of the stronger expression level of the integrin αVβ5 receptor on TCs, we further verified the beneficial effect of irisin on TCs. Irisin can also enhance the viability of DHT-induced TCs, and this effect was even more pronounced than in GCs (Fig. 6A). Similarly, irisin markedly inhibited the activation of the IRE1α-TXNIP/ROS-NLRP3 signaling pathway (Fig. 6B-G). Taken together, these observations suggested that irisin could improve the viability of ovarian cells, which may be mediated through the IRE1α-TXNIP/ROS-NLRP3 signaling pathway.

### Irisin inhibits inflammasome activation and fibrosis in GCs and TCs

**Fig. 7:**
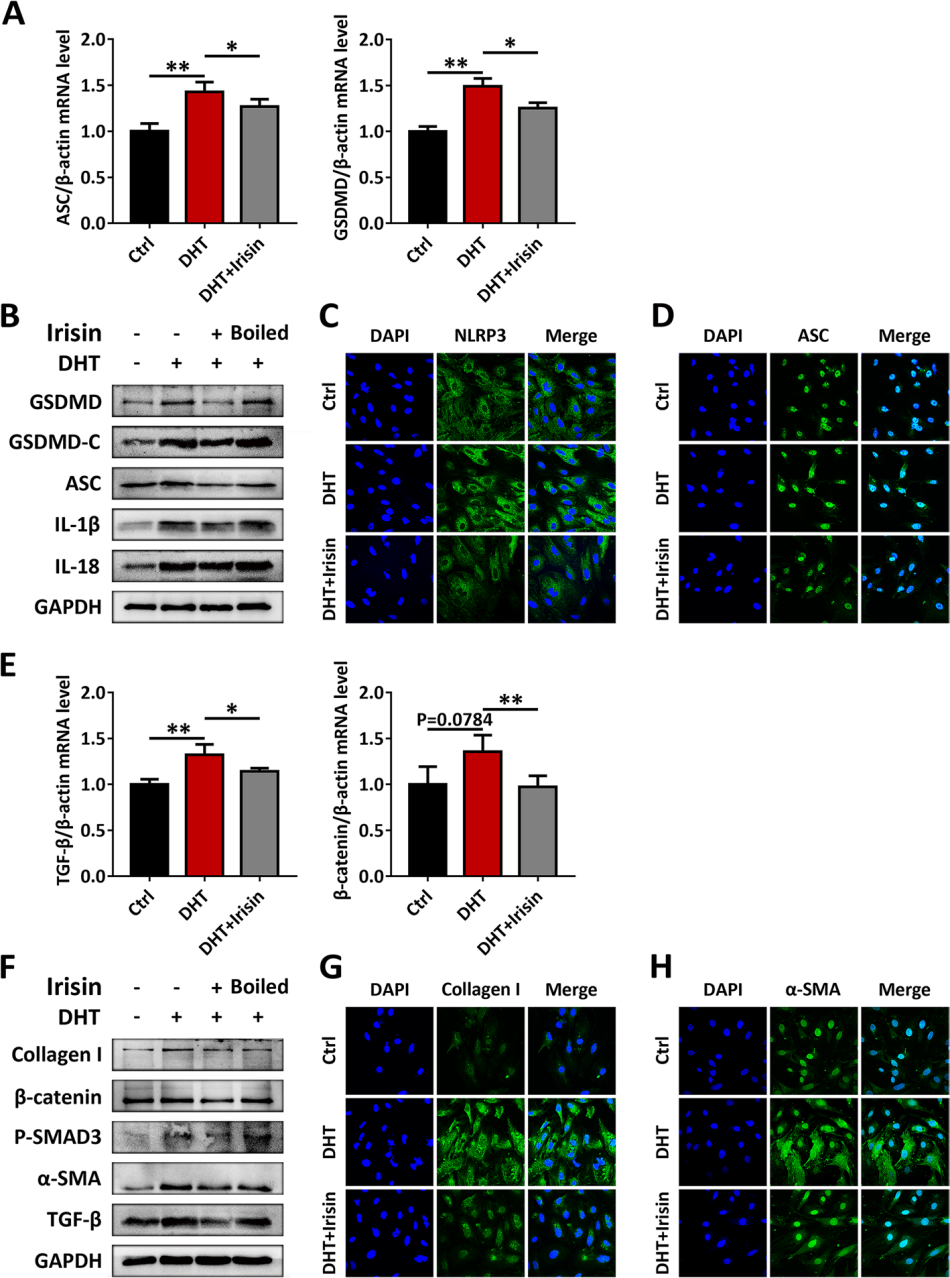
Irisin inhibits GCs inflammasome activation and fibrosis. GCs were treated with DHT, in the presence or absence of irisin. **A** The expression of ASC and GSDMD in GCs was assessed by qRT-PCR. **B** The expression of NLRP3 inflammasome activation factors (GSDMD, GSDMD-C, ASC, IL-1β and IL-18) in GCs was analyzed by western blot assay. **C-D** The protein levels of NLRP3 (**C**) and ASC (**D**) in GCs were analyzed by immunofluorescence staining (60 ×). (**E**) The expression of TGF-β and β-catenin in GCs was assessed by qRT-PCR. **F** The expression of fibrosis factors (collagen I, β-catenin, P-SMAD3, α-SMA, TGF-β) in GCs was analyzed by western blot assay. **G-H** The protein levels of collagen I (**G**) and α-SMA (**H**) in GCs were analyzed by immunofluorescence staining (60 ×). Data are shown as the mean ± SD. *p ≤ 0.05, **p ≤ 0.01. Ctrl, control; DHT, dihydrotestosterone; ASC, apoptosis-associated speck-like protein containing a CARD; GSDMD, gasdermin D; GSDMD-C, C-terminal fragment of gasdermin D; IL-1β, interleukin-1β; IL-18, interleukin-18; GAPDH, glyceraldehyde-3-phosphate dehydrogenase; DAPI, 4’,6-diamidino-2-phenylindole; NLRP3, NOD-like receptor family pyrin domain containing 3; TGF-β, transforming growth factor-beta; α-SMA, a-smooth muscle actin

**Fig. 8:**
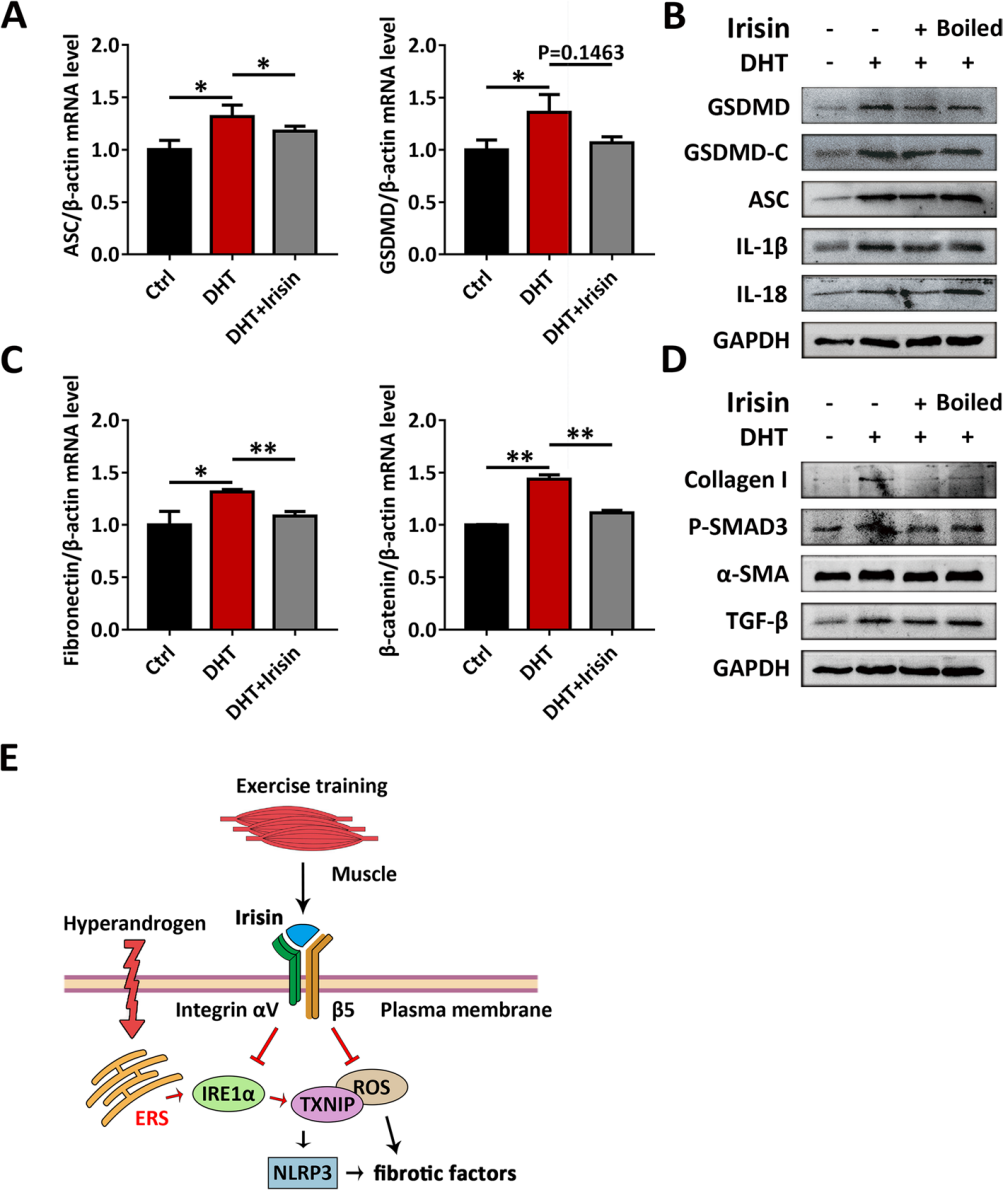
Irisin inhibits TCs inflammasome activation and fibrosis. TCs were treated with DHT, in the presence or absence of irisin. **A** The expression of ASC and GSDMD in TCs was assessed by qRT-PCR. **B** The expression of NLRP3 inflammasome activation factors (GSDMD, GSDMD-C, ASC, IL-1β and IL-18) in TCs was analyzed by western blot assay. **C** The expression of fibronectin and β-catenin in TCs was assessed by qRT-PCR. **D** The expression of fibrosis factors (collagen I, P-SMAD3, α-SMA, TGF-β) in TCs was analyzed by western blot assay. **E** Exercise-induced irisin improves follicular dysfunction by inhibiting the IRE1α-TXNIP/ROS-NLRP3 pathway in the PCOS signaling pathway. Data are shown as the mean ± SD. *p ≤ 0.05, **p ≤ 0.01. Ctrl, control; DHT, dihydrotestosterone; ASC, apoptosis-associated speck-like protein containing a CARD; GSDMD, gasdermin D; GSDMD-C, C-terminal fragment of gasdermin D; IL-1β, interleukin-1β; IL-18, interleukin-18; GAPDH, glyceraldehyde-3-phosphate dehydrogenase; α-SMA, a-smooth muscle actin; TGF-β, transforming growth factor-beta; ERS, endoplasmic reticulum stress; ROS, reactive oxygen species; IRE1α, inositol-requiring enzyme 1α; TXNIP, thioredoxin-interacting protein; NLRP3, NOD-like receptor family pyrin domain containing 3

Finally, we tried to confirm whether irisin could improve DHT-induced inflammasome activation and fibrosis. The mRNA levels of inflammasome activation factors ASC and GSDMD in GCs were significantly reduced following treatment with irisin (Fig. 7A). The protein levels of GSDMD, GSDMD-C, ASC, IL-1β and IL-18 were also markedly reduced, and this treatment effect was abrogated if irisin was boiled prior to the treatment (Fig. 7B). The decreased expression of NLRP3 and ASC after irisin pretreatment was also confirmed by immunofluorescence staining (Fig. 7C-D). Similarly, after irisin pretreatment, the mRNA levels of the hyperandrogen-induced fibrosis factors TGF-β and β-catenin were also decreased (Fig. 7E). The protein levels of collagen I, β-catenin, P-SMAD3, α-SMA, and TGF-β were decreased after pretreatment with native irisin, but not boiled irisin (Fig. 7F). Immunofluorescence staining confirmed that the expression of collagen I and α-SMA was decreased after irisin pretreatment (Fig. 7G-H). Similarly, we also confirmed that irisin pretreatment inhibited the expression of inflammasomes and fibrotic factors in TCs (Fig. 8A-D).

Our data strongly argues for the beneficial effect of irisin as an exercise-induced myokine. It could remarkably improve the viability of GCs and TCs exposed to hyperandrogen and suppress the activation of inflammasomes, fibrosis and OS. This effect may be mediated by inhibition of the IRE1α-TXNIP/ROS-NLRP3 signaling pathway (Fig. 8E).

## Discussion

PCOS is assumed to be caused and modulated by multiple factors. Women with PCOS often have hyperandrogenism, chronic inflammation, obesity, insulin resistance and abnormal lipid metabolism [37]. Research on PCOS has advanced considerably in recent years, but its exact pathogenesis remains elusive. We have previously found that the activation of NLRP3 inflammasomes and thus enhanced ovarian fibrosis are important causal factors of PCOS ovarian dysfunction [18], but its upstream regulatory networks remain to be elucidated.

The ER plays an important role in regulating various intracellular physiological functions, including protein transport, protein folding, calcium homeostasis, and lipid biosynthesis. The homeostasis of ER will be disturbed under physiological and pathophysiological conditions. Accumulation of unfolded or misfolded proteins in the lumen of the ER can lead to ERS. When stress occurs in the ER, the BIP, also known as 78-kD glucose-regulated protein (GRP78), is separated during signal transduction and stress responses, followed by preferential binding to unfolded or misfolded proteins. These events will lead to the activation of three distinct ER transmembrane sensor proteins: IRE1α, pancreatic endoplasmic reticulum kinase (PERK), and activating transcription factor 6 (ATF6), which are capable of initiating complex UPR signaling, thus leading to apoptosis, inflammation, and OS [38]. Previous studies have shown that ERS can activate the NLRP3 inflammasome through OS. ERS can lead to enhanced production of ROS and release of TXNIP from TRX, resulting in the binding and activation of the NLRP3 inflammasome. In addition, IRE1α can also promote the activation of the NLRP3 inflammasome by inhibiting miR-17-5p [21]. Our study shows that the expression of IRE1α protein was significantly increased in the ovaries of PCOS rats, suggesting that ERS could be associated with the pathogenesis of PCOS. IRE1α siRNA markedly decreased the total amount of IRE1α, and the expression of inflammasome activity and fibrotic factors in primary ovarian cells following exposure to DHT was abrogated. Taken together, these data support an important role of IRE1α in hyperandrogen-induced ovarian dysfunction.

The etiology of PCOS is complex and diverse, and there are currently no therapeutic approaches that target the pathogenetic mechanisms. The management of PCOS usually focuses on symptomatology such as infertility or hirsutism. Exercises can be considered an attractive therapeutic intervention for this chronic disease because of the low cost and low threshold. Two studies by Selvaraj et al. found that two months of yoga and two months of walking exercise can lower the risk of PCOS [10, 11]. Work by Hansen et al. points out that 14 weeks of supervised exercise training can improve hyperandrogenemia in women with PCOS [12]. Wu et al. found that 12 weeks of aerobic exercise have a beneficial effect on BMI, cardiovascular health, AMH level and the degree of OS in women with PCOS [13]. After 16 weeks of continuous or intermittent aerobic physical exercise, the anthropometric indicators of women with PCOS have improved, and serum androgen levels are decreased. In addition, continuous aerobic physical exercise can improve the blood lipid status of women with PCOS [14]. Several studies have also shown that exercise has a positive effect on reproduction and is associated with improvements in insulin sensitivity and visceral lipid metabolism [39–41]. Exercise for 12 to 24 weeks can increase the ovulation rate and insulin sensitivity and facilitate weight loss [42]. In this study, we found that eight weeks of flat treadmill exercise training could effectively improve the ovarian morphology, serum sex hormone regulation and ovarian function of PCOS rats. The NLRP3 inflammasome activation and fibrosis were also alleviated. However, the specific molecular mechanisms underlying exercise-mediated improvement of PCOS symptoms remain to be further explored.

Irisin, a novel myokine induced by exercise, was identified by Bostrom et al. in 2012 [25]. Irisin is a polypeptide fragment that is cleaved and modified by FNDC5 and secreted into the blood, and it can promote the transition of white adipose tissue to brown adipose tissue. Brown adipose tissue contains large amounts of mitochondrial protein uncoupling protein-1 (UCP-1), which can convert energy produced by mitochondria to thermal energy, promote energy expenditure, and thus play a role in regulating energy metabolism. Furthermore, irisin was also reported to increase glucose uptake and the expression of glucose transporter 4 (GLUT4). Exogenous irisin treatment can significantly improve insulin resistance in high-fat diet-fed rats. In addition, irisin can target the mitochondria of damaged cells in organs with ischemia–reperfusion injury, inhibit the production of ROS and the activation of inflammatory factors caused by ischemia–reperfusion, control the formation of harmful free radicals, and reduce the OS response. Interestingly, several recent studies have suggested beneficial effects of irisin on inflammasome activation and fibrosis. In 2018, Kim first discovered the irisin receptor αV integrin in fat and bone cells [36]. Since then, integrin αVβ5 has been confirmed to be the irisin receptor in a number of studies. In this study, we could show that integrin αVβ5 was strongly expressed in rat ovarian TCs and moderately expressed in GCs using immunofluorescence. Consequently, using ex vitro primary ovarian TC and GC models, we found that irisin could reverse DHT-induced activation of the IRE1α-TXNIP/ROS-NLRP3 pathway and inhibit the expression of fibrosis factors. As a result, irisin exhibits beneficial effects of counter hyperandrogen in ovarian cells, whether irisin will effectively improve PCOS merited further research. Exogenous myokines may play an important role in motor function and movement and predicted to be a potential drug for the treatment and prevention of chronic diseases. The translational significance of the current study lies in the fact that a scientifically rational exercise evaluation system for clinical treatment may be designed based on the findings in our rodent models. The limitation of this study includes the lack of in vivo confirmation of the therapeutic function of irisin. The mechanism underlying the inhibitory role of irisin in the IRE1α-TXNIP/ROS-NLRP3 pathway awaits further clarification.

## Conclusion

In conclusion, the current study provides both in vivo and in vitro evidence demonstrating that long-term aerobic exercise training (prescription exercise) can improve PCOS-disrupted oestrous cycles, polycystic ovaries and reproductive hormone variations. Precisely, such exercise was shown to facilitate the restoration of ovarian morphology and reduction of ovarian inflammation, OS and fibrosis in PCOS-like rats. We further revealed that hyperandrogen promoted ROS production by inducing ERS in GCs and TCs, activated the IRE1α-TXNIP/ROS-NLRP3 signaling pathway, and promoted NLRP3 inflammasome activation, which in turn lead to GC dysfunction and follicular development disorders. Exercise-induced myokine irisin could exert anti-inflammatory and anti-OS effects, and inhibit the activation of the IRE1α-TXNIP/ROS-NLRP3 signaling pathway in the PCOS ovary, thereby improving the ovarian function of PCOS-like rats induced by hyperandrogen.

## Supplementary Information

**Additional file 1:** **Supplementary Figure 1.** IRE1α silencing by siRNA rescues DHT-induced dysfunction of TCs.

**Additional file 2: Supplementary Figure 2.** Exercise reduces the level of ovarian fibrosis in PCOS rats.

## Data Availability

The data that support the findings of this study are available from the corresponding author upon reasonable request.

## Abbreviations

AF: Antral follicle
ANOVA: One-way analysis of variance
ASC: Apoptosis-associated speck-like protein containing a CARD
AR: Androgen receptor
ATF6: Activating transcription factor 6
α-SMA: A-smooth muscle actin
αVβ5: Integrin αVβ5
BIP: ER chaperone binding immunoglobulin protein
BMP: Bone morphogenetic protein
BSA: Bovine serum albumin
CCK8: Cell Counting Kit-8
CF: Cystic follicles
CL: Corpus luteum
Ctrl: Control
CYP11α1: Cytochrome p450 11
CYP19α1: Cytochrome p450 19
DAPI: 4’,6-Diamidino-2-phenylindole
DCF-DA: Dichlorofluorescein diacetate
DHEA: Dehydroepiandrosterone
DHT: Dihydrotestosterone
D + E: DHEA + exercise
ELISA: Enzyme-linked immunosorbent assay
ER: Endoplasmic reticulum
ERS: Endoplasmic reticulum stress
H&E: Hematoxylin and eosin
FBS: Fetal bovine serum
FGF-2: Fibroblast growth factor-2
FNDC5: Fibronectin type III domain-containing protein-5
FSH: Follicle stimulating hormone
GAPDH: Glyceraldehyde-3-phosphate dehydrogenase
GCs: Granulosa cells
GLUT4: Glucose transporter 4
GRP78: 78-KD glucose-regulated protein
GSDMD: Gasdermin D
GSDMD-C: C-terminal fragment of gasdermin D
GSDME: Gasdermin E
IGF-1: Insulin-like growth factor-I
IL-18: Interleukin-18
IL-6: Interleukin-6
IL-7: Interleukin-7
IL-15: Interleukin-15
IL-1β: Interleukin-1β
IRE1α: Inositol-requiring enzyme 1α
LH: Luteinizing hormone
MDA: Malondialdehyde
NC: Normal contrast
NLRP3: NOD-like receptor family pyrin domain containing 3
OGN: Osteoglycin
OS: Oxidative stress
PAF: Preantral and early antral follicle
PCOS: Polycystic ovary syndrome
PERK: Pancreatic endoplasmic reticulum kinase
PGC-1α: Peroxisome proliferator-activated receptor-γ coactivator-1α
PMSG: Pregnant mare serum gonadotropin
ROS: Reactive oxygen species.
SD: Sprague–Dawley.
SEM: Means ± standard error of the mea.
si: SiRNA.
siRNA: Small interfering RNA.
SOD: Superoxide dismutase.
SPF: Specific pathogen-free.
TCs: Theca cells.
TGF-β: Transforming growth factor-beta.
TRX: Thioredoxin.
TXNIP: Thioredoxin-interacting protein.
UCP-1: Uncoupling protein-1.
UPR: Unfolded protein response
