# Supplementary figures and images for: Exercise-induced irisin improves follicular dysfunction by inhibiting IRE1α-TXNIP/ROS-NLRP3 pathway in PCOS
Source: J Ovarian Res. 2023 Jul 31;16:151. doi: 10.1186/s13048-023-01242-x (PMC10388501; doi:10.1186/s13048-023-01242-x)

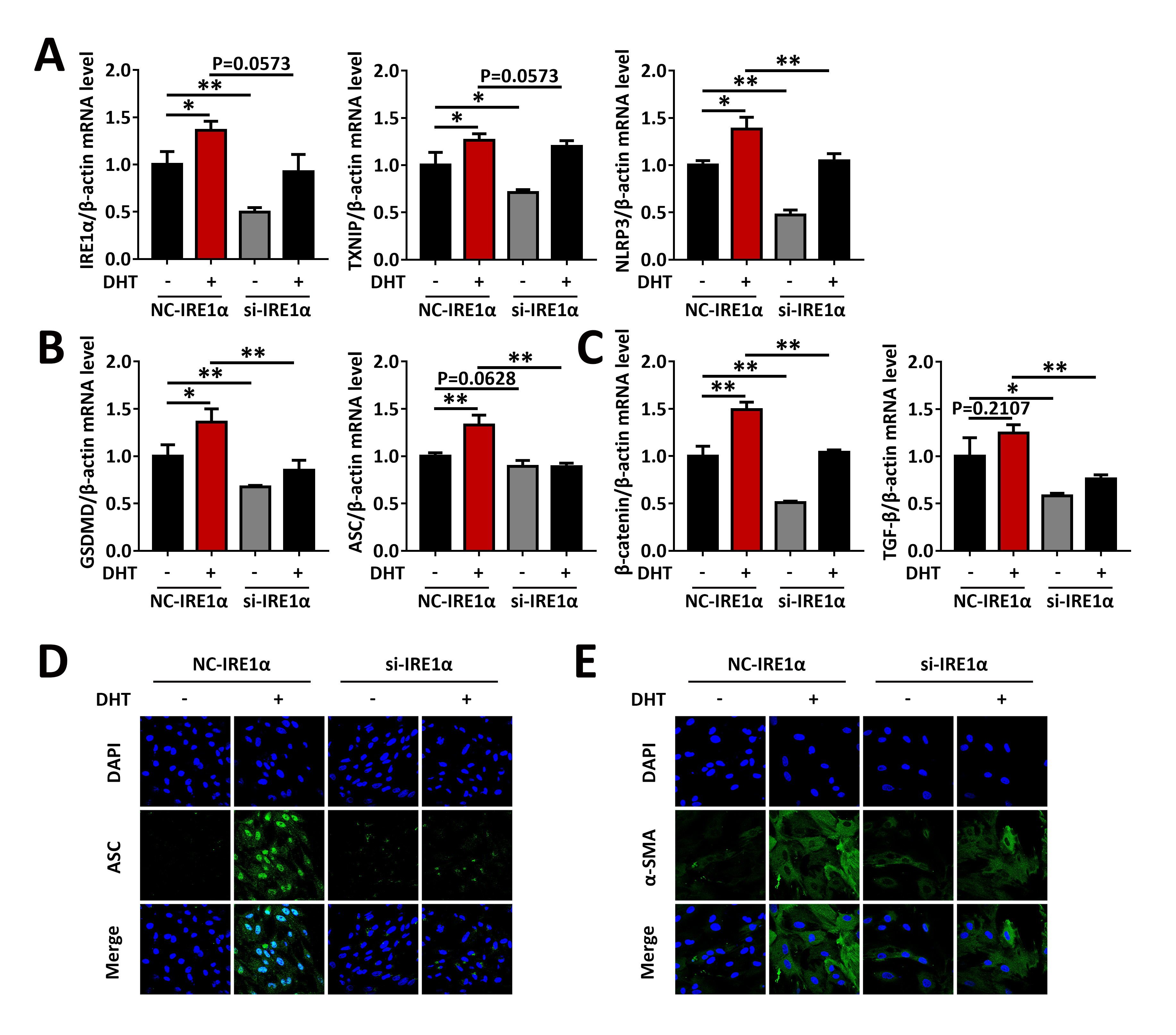

Supplement: Supplementary file 1 — Additional file 1: Supplementary Figure 1. IRE1α silencing by siRNA rescues DHT-induced dysfunction of TCs. [file 13048_2023_1242_MOESM1_ESM.jpg]

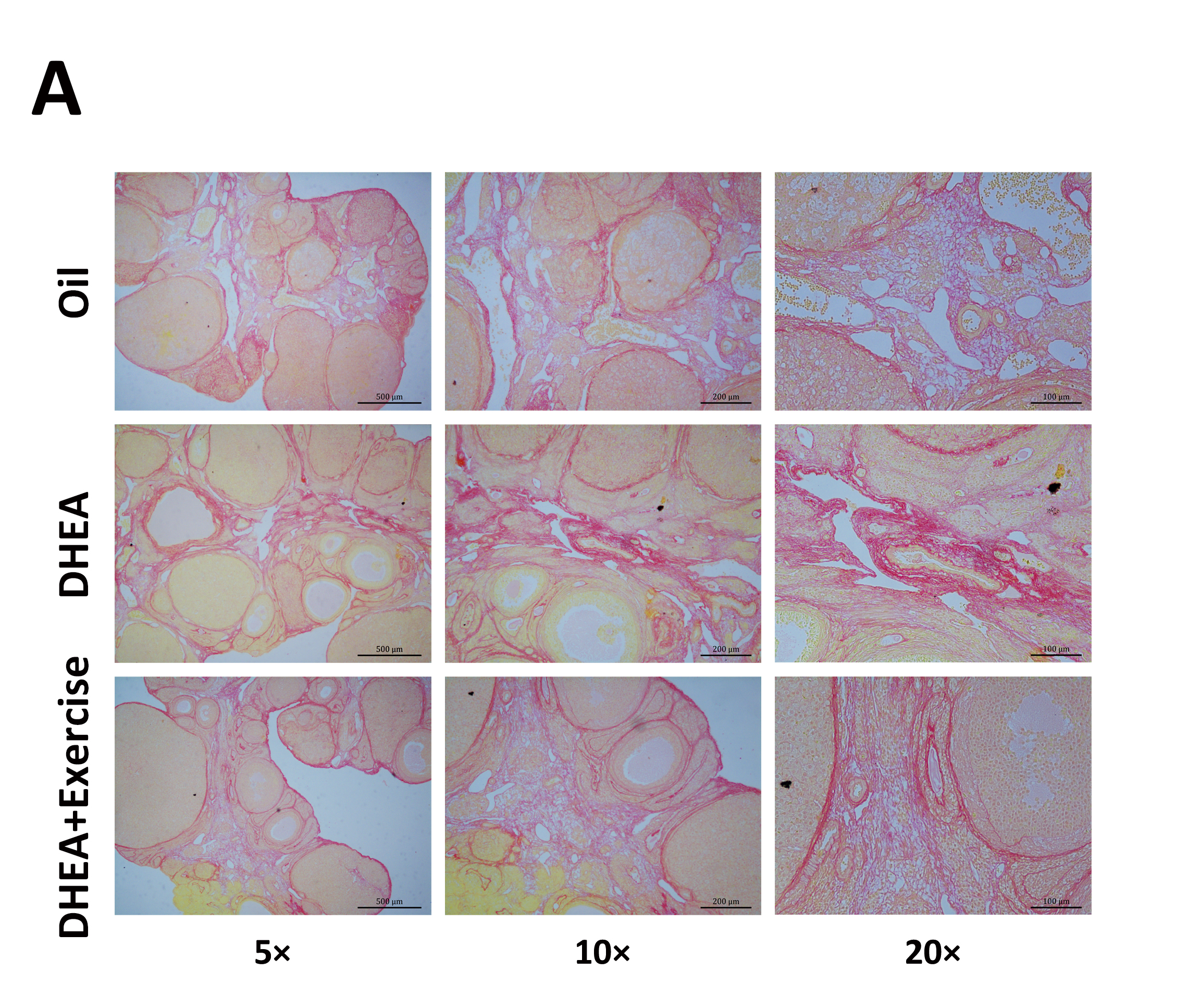

Supplement: Supplementary file 2 — Additional file 2: Supplementary Figure 2. Exercise reduces the level of ovarian fibrosis in PCOS rats. [file 13048_2023_1242_MOESM2_ESM.jpg]
